# *SPINK4* modulates inhibition of glycolysis against colorectal cancer progression

**DOI:** 10.17305/bb.2024.10338

**Published:** 2024-12-01

**Authors:** Xiaodi Yang, Sen Jiang, Zhen Yuan, Jun Jiang, Mengxuan Yang, Jing Luo, Tao Ye

**Affiliations:** 1 Department of Oncology, Minhang Branch, Zhongshan Hospital, Fudan University, Shanghai, China; 2 Key laboratory of Whole-Period Monitoring and Precise Intervention of Digestive Cancer (SMHC), Minhang Hospital and AHS, Fudan University, Shanghai, China; 3 Department of Internal Emergency Medicine, Shanghai East Hospital, Tongji University School of Medicine, Shanghai, China; 4 Shanghai East Clinical Medical College, Nanjing Medical University, Shanghai, China; 5 State Key Laboratory of Genetic Engineering, Shanghai Engineering Research Center of Industrial Microorganisms, School of Life Sciences, Fudan University, Shanghai, China

**Keywords:** Serine peptidase inhibitor, Kazal type 4 (SPINK4), glycolysis, colorectal cancer (CRC), prognosis

## Abstract

Dysregulation of glycolysis is frequently linked to aggressive tumor activity in colorectal cancer (CRC). Although serine peptidase inhibitor, Kazal type 4 (*SPINK4*) has been linked to CRC, its exact linkage to glycolytic processes and gene expression remains unclear. Differentially expressed genes (DEGs) were screened from two CRC-related datasets (GSE32323 and GSE141174), followed by expression and prognostic analysis of *SPINK4*. In vitro techniques, such as flow cytometry, western blotting (WB), transwell assay and quantitative real-time polymerase chain reaction (qRT-PCR) were used to assess *SPINK4* expression in CRC cells. Its effects on apoptosis, glycolysis, and the cell cycle were also investigated. Finally, the impact of *SPINK4* overexpression on tumor development was assessed using a xenograft model, while histological and immunohistochemical analyses characterized SPINK4 expression patterns in CRC tissues. *SPINK4* expression was downregulated in CRC, correlating with poor patient prognosis. In vitro assays confirmed that overexpression of *SPINK4* reduced CRC cell proliferation, invasion, and migration, while its knockdown promoted these processes and caused G1 arrest. *SPINK4* also regulated apoptosis by altering caspase activation and Bcl-2 expression. Besides, *SPINK4* overexpression altered glycolytic activity, reduced 2-Deoxy-D-glucose (2-DG) absorption, and controlled critical glycolytic enzymes, resulting in alterations in metabolic pathways, whereas *SPINK4* knockdown reversed this effect. *SPINK4* overexpression significantly reduced tumor volume in vivo, indicating its inhibitory role in carcinogenesis. Moreover, high expression of SPINK4, hexokinase 2 (HK2), glucose transporter 1 (GLUT1), lactate dehydrogenase A (LDHA), and pyruvate kinase M2 (PKM2) was observed in CRC tissues. As a key inhibitor of glycolytic metabolism in CRC, *SPINK4* promises metabolic intervention in CRC therapy due to its impact on tumor growth and cell proliferation.

## Introduction

Colorectal cancer (CRC) is one of the common malignant tumors of the digestive tract and originates from the transformation of adenomatous polyps [1, 2]. Its complicated etiology involves genetic, environmental, and lifestyle factors [3]. Causative factors include age, family history, certain genetic abnormalities, and inflammatory bowel disease [4]. Despite improvements in treatment approaches and early detection, CRC remains a significant global health concern because of its high morbidity and mortality [5]. The need for targeted preventative measures and improved treatment options is highlighted by the variations in incidence and death rates among regions [6]. Presently, the objectives of CRC research include elucidating the molecular pathways of tumor genesis and development and developing new prognostic indicators, diagnostic markers, and therapeutics [7, 8]. This is essential for formulating personalized treatment regimens and developing innovative precision medicine techniques, as well as for gaining a thorough understanding of the pathophysiology of CRC [9].

Glycolysis is a basic metabolic process in cells that arranges the breakdown of glucose into smaller molecules and is necessary for the production of adenosine triphosphate (ATP) [10]. Glycolysis is a cytoplasmic process that starts with the phosphorylation of glucose and proceeds to fructose-1,6-bisphosphate, which is subsequently converted to glyceraldehyde-3-phosphate and dihydroxyacetone phosphate [11]. Further reactions with these three-carbon molecules produce pyruvate, nicotinamide adenine dinucleotide (NADH), and ATP [12]. Remarkably, glycolysis, operating independently of oxygen, initiates both aerobic and anaerobic cellular respiration. The end products, like pyruvate, adapt to various metabolic pathways based on cellular conditions. In the context of CRC, glycolysis plays a critical role. According to a study by Zuo et al. [13], vitamin D-activated long non-coding RNA MEG3 reduces glycolysis in CRC via encouraging c-Myc degradation, pointing to vitamin D as a possible treatment target. Additionally, the study by Zhu et al. [14] linked tumorigenesis in Dectin-3-lacking mice to commensal fungus *Candida albicans*, emphasizing the interconnected roles of macrophage-induced glycolysis and interleukin-22 secretion by innate lymphoid cells in CRC, thus emphasizing the influence of the fungal flora. Additionally, the work by Zhu et al. [15] emphasized the crucial function of *PTBP1* in CRC as a critical regulator of glycolysis and other important cellular processes. Thus, a thorough understanding of glycolysis is crucial for CRC research, as it is for defining cellular metabolic pathways and developing targeted therapeutics for a variety of illnesses.

Serine peptidase inhibitor, Kazal type 4 (*SPINK4*) is a gene found on chromosome 19 of the human genome. Its protein product is an important regulator of serine proteases engaged in several physiological processes, and it belongs to the family of serine protease inhibitors (Kazal type) [16]. Although the precise roles of *SPINK4* are unidentified, it is usually linked to the inhibition of serine peptidases, which are vital enzymes involved in the degradation of proteins. Notably, *SPINK4* has led to investigations into its expression levels and regulatory role in cellular processes in cancer research, particularly in CRC [17]. According to the research of Xie et al. [18], increasing serum *SPINK4* levels in CRC, particularly in patients who are about to undergo surgery, indicate diagnostic potential with improved sensitivity and specificity. The higher SPINK4 levels are related to the advancement of CRC as they correspond with the site of cancer and distant metastases. According to an analysis of comprehensive gene expression, survival data, and immunohistochemistry validation, research by Wang et al. [19] showed that downregulated SPINK4 expression is an independent predictor of reduced survival in CRC patients. Moreover, alpha-1 antitrypsin (A1AT) and elevated genes *REG4* and *SPINK4* are related to cancer-associated thrombosis (CAT) in CRC according to research by Buijs et al. This suggests that these genes might be used as biomarkers to identify CRC patients [20]. In addition to opening up new possibilities for diagnostic and treatment approaches, an understanding of the roles and regulation of *SPINK4* offers insightful insights into the molecular mechanisms behind the development and progression of cancer in CRC.

This study investigated the impact of *SPINK4* on glycolytic metabolism and CRC cell proliferation to investigate the critical role of glycolysis and the importance of this biomarker for CRC. The effects of *SPINK4* expression on the prognosis, glycolytic enzyme levels, cellular energy balance, and cell cycle of CRC patients will be clarified in turn below. By integrating these molecular findings, we aim to improve our understanding of CRC pathophysiology and provide fresh perspectives into targeted therapeutic strategies.

## Materials and methods

### Download and processing of GSE32323 and GSE141174 datasets

Two CRC-related datasets, namely, GSE32323 and GSE141174, were obtained from the Gene Expression Omnibus (GEO, https://www.ncbi.nlm.nih.gov/gds/) repository. The GSE32323 dataset consisted of 44 samples, of which 17 tumor samples were chosen as the case group and 17 normal samples as the control group. The GSE141174 dataset includes 16 samples, from which three CRC primary tumor samples were selected as the case group and three normal samples were selected as the control group. The Limma package in R language was used for the differential gene expression study. Fold changes (FCs) greater than 2 were considered upregulated differentially expressed genes (DEGs), whereas genes with an FC less than 0.5 were regarded as downregulated DEGs. The significance criterion for the *P* value was set at < 0.05.

### Survival analysis of differential expression of SPINK4

The Bioinformatic platform (http://www.bioinformatics.com.cn/) was used to analyze the intersection DEGs in the GSE32323 and GSE141174 datasets, including *SPINK4*, and visualized through the Venn diagram. Next, in various groups of the GSE32323 and GSE141174 datasets, the differential expression of *SPINK4* was compared. The effect of the differential expression of *SPINK4* on the overall survival (OS) probability of CRC patients was then assessed using the Kaplan–Meier plotter (https://kmplot.com/analysis/), and the significance of the difference was determined using the log-rank test.

### Cell lines and culture

The Chinese Academy of Sciences Cell Collection Center (Shanghai, China) provided four CRC cell lines (LoVo, HCT-116, RKO, and SW480) as well as normal colorectal epithelial cells (NCM460). After that, the cells were cultivated in DMEM and kept at 37 ^∘^C in a humidified atmosphere with 5% CO_2_. DMEM was supplemented with 10% fetal bovine serum (FBS) and 1% penicillin–streptomycin.

### Cell transfection

For transient transfection, 2 × 10^5^ CRC cells were planted per well in 24-well plates. Transfection of the plasmid encoding *SPINK4* into CRC cells using an appropriate transfection method will allow the transfected cells to express the *SPINK4* protein for a specific period to achieve overexpression. Then, two specific small interfering RNAs (siRNAs), si-*SPINK4*-1 and si-*SPINK4*-2, were introduced into the CRC cells to achieve knockdown of *SPINK4* expression, and cells were incubated for a specific time to allow efficient knockdown of *SPINK4*. Cells were transfected using Lipofectamine 3000 (Invitrogen, USA) by the manufacturer’s instructions.

### Cell treatment

CRC cells were seeded at a density of 2 × 10^5^ cells/well in 24-well plates. After 24 h of incubation to facilitate cell adhesion, cells underwent treatment with different substances for 48 h as part of the specific study. Treatments include 0.5 mM Glucose (Glu), to assess basic cellular metabolic responses; 20 µM Cytochalasin B (Cyto-B), to study effects on cytoskeletal dynamics and cell motility; 10 µg/mL 3-Bromopyruvate (3-BrPA), to study the effects on glycolytic pathways and energy metabolism; 50 µg/mL 5-Fluorouracil (5-FU), to investigate the impact of chemotherapy on the survival and proliferation of cells; and 10 µM XAV −939 to examine the effects on Wnt/β-catenin signaling and related cellular processes. Without any active ingredient, cells in the control group were treated with an equivalent amount of dimethyl sulfoxide (DMSO). After treatment, cells were subjected to subsequent analysis.

### Quantitative real-time polymerase chain reaction (qRT-PCR) assay

The TRIzol reagent (Thermo Fisher Scientific, USA) was used to extract the total RNA of CRC cells by following the manufacturer’s instructions. For cDNA synthesis, we utilized a PrimeScript RT kit (Takara, Japan). SYBR Green PCR Master Mix (Applied Biosystems, USA) was used to perform qRT-PCR using the StepOnePlus Real-Time PCR System (Applied Biosystems, USA). Gene expression levels of *SPINK4*, β-catenin, pyruvate kinase M2 (*PKM2*), glucose transporter 1 (*GLUT1*), lactate dehydrogenase A (*LDHA*), and hexokinase 2 (*HK2*) were quantified and normalized to *GAPDH*. All target expression levels were computed with the 2^−ΔΔCT^ technique. A set of primer sequences was found in Table 1.

**Table 1:** Primer sequences for qRT-PCR

**Target**	**Direction**	**Sequence**
SPINK4	Forward	5′-GACATTTCAGGGAGGGGACA-3′
SPINK4	Reverse	5′-TGGTTCCCTGTCCTGATCAC-3′
β-catenin	Forward	5′-AAAGCGGCTGTTAGTCACTGG-3′
β-catenin	Reverse	5′-CGAGTCATTGCATACTGTCCAT-3′
PKM2	Forward	5′-GACTGCCTTCATTCAGACCCA-3′
PKM2	Reverse	5′-GGGTGGTGAATCAATGTCCAG-3′
GLUT1	Forward	5′-CGGGCCAAGAGTGTGTGCTAAA-3′
GLUT1	Reverse	5′-TGACGATACCGGAGCCAATG-3′
LDHA	Forward	5′-GGCCTGTGCCATCAGTATCT-3′
LDHA	Reverse	5′-GGAGATCCATCATCTCTCCC-3′
HK2	Forward	5′-CCAGTTCATTCACATCATCAG-3′
HK2	Reverse	5′-CTTACACGAGGTCACATAGC-3′
GAPDH	Forward	5′-CAAGCTCATTTCCTGGTATGAC-3′
GAPDH	Reverse	5′-CAGTGAGGGTCTCTCTCTTCCT-3′

qRT-PCR: Quantitative real-time polymerase chain reaction; SPINK4: Serine peptidase Inhibitor, Kazal type 4.

### Western blotting (WB) assay

Protease and phosphatase inhibitors were added to the RIPA lysis solution (Thermo Fisher Scientific, USA) to produce protein lysates from CRC cells. The BCA Protein Assay Kit (Thermo Fisher Scientific, USA) was utilized to ascertain the protein content. Proteins in equal quantities were separated using 10% SDS-PAGE and then put onto PVDF membranes from Millipore, USA. After membrane sealing with 5% skimmed milk, primary antibodies targeting SPINK4, TP53, Cyclin D1, P21, P18, CDK4, CDK6, Caspase 3, Caspase 9, Bcl-2, β-catenin, PKM2, GLUT1, LDHA, and HK2 were applied at a 1:1000 dilution for incubation. After that, the membranes were incubated with suitable horseradish peroxidase (HRP)-conjugated secondary antibodies. As an internal reference, GAPDH (1:5000) was employed. Thermo Fisher Scientific, USA, provided an enhanced chemiluminescence (ECL) kit for visualizing protein bands, and Bio-Rad, USA, provided ChemiDoc imaging equipment for capturing the images.

### Cell viability assay

The viability of the cells was assessed using the cell counting kit-8 (CCK-8) assay (Dojindo, Japan). CRC cells were added at a density of 5 × 10^3^ cells per well in 96-well plates. After treatment, each well was filled with CCK-8 reagent, and the absorbance at 450 nm was measured using a microplate reader (Thermo Fisher Scientific, USA) at 24, 48, 72, 96, and 120 h. Additionally, a focused analysis at the 48-h mark was conducted to determine the effects of different treatments on cell viability. Treatments included exposure to 0.5 mM Glu, 20 µM/L Cyto-B, and 10 µg/mL 3-BrPA.

### Transwell assays

The Transwell system was used to assess cell invasion and migration. Transfected CRC cells were placed in the upper chamber of the Transwell using serum-free media. This was followed by the addition of 10% FBS to the medium in the lower chamber of the Transwell. After a designated incubation period, cells with motile membranes were stained with crystal violet and fixed with 4% paraformaldehyde. Lastly, the quantity of migrating cells in the field of view was counted using inverted microscopy. The cell invasion studies were carried out as described previously, with matrigel covered in the upper chamber.

### Flow cytometry

CRC cells were initially collected using trypsin-ethylenediaminetetraacetic acid (EDTA) (Gibco, USA) and rinsed in phosphate-buffered saline (PBS) to undertake apoptosis and cell cycle analysis. The manufacturer’s instructions were followed while staining the cells with Annexin V and propidium iodide (PI) to detect apoptosis and distinguish between viable, apoptotic, and necrotic cells. Cells were frozen in ethanol, subjected to RNase A treatment, and stained with PI to analyze the DNA content to evaluate the cell cycle. Evaluation of the patterns of cell death and proliferation was made easier by these approaches. Flow cytometry was performed using a BD Biosciences instrument, and FlowJo software was used for data analysis.

### Glucose uptake assay

To assess glucose uptake, 2-DG was employed as a glucose tracer. Briefly, a 6-well plate was seeded with around 1 × 10^5^ cells in quadruplicate, and the plate was then incubated at 37 ^∘^C with 5% CO_2_ for an entire night. On the following day, cells were subjected to a 4-h glucose deprivation, followed by a 2-h incubation with 25 µM 2-DG for three wells and 25 µM glucose-containing CRC medium for the remaining well as a negative control. Cells were taken out of the incubation phase and given two PBS washes. The cellular uptake of 2-DG was quantified by measuring the average fluorescence intensity of the cells using flow cytometry with excitation light.

### Lactate and ATP production measurements

CRC cells were plated at a density of 1 × 10^5^ cells per well in 24-well plates for lactate assays and 1 × 10^4^ cells per well in 96-well plates for ATP assays to quantify the generation of lactate and ATP. The cells were seeded in triplicate for lactate assays and quintuplets for ATP assays and allowed to incubate for 24 h. After the first incubation, RPMI 1640 with 1 mM Glu was added to the media and left overnight. The culture media was taken for the ATP and lactate tests the next day. Lactate concentration was measured using commercial assay kits (Abcam) according to the manufacturer’s instructions. Simultaneously, relative ATP concentration was measured using commercial assay kits (Abcam). This combined approach allowed for the comprehensive assessment of both lactate and ATP production in CRC cells under the specified conditions.

### Xenograft model

Four-week-old naked mice had their flanks subcutaneously implanted with 1.0 × 10^6^ stable transfected HCT-116 cells. Upon the onset of visible tumors in every animal following ten days of subcutaneous injection, the drug delivery experiments were initiated. The progression of tumor growth was monitored systematically, and the standard formula, tumor volume ═ (*L* × *W*^2^)/2, was used to calculate the tumor volume (mm^3^), with *W* representing the width and *L* representing the length of the tumor. Harvesting of tumors was executed upon reaching an approximate diameter of 2.0 cm.

### Histological staining

Tumor samples were fixed in 4% paraformaldehyde and embedded in paraffin wax. The embedded samples were sectioned into 4 µm slices for staining and microscopic observation. Following complete deparaffinization, the sections underwent Hematoxylin and Eosin (HE) staining. Specifically, the sections were stained with Hematoxylin for 5 min using a Hematoxylin solution (ZSGB-BIO, China). Subsequently, 1% acidic ethanol was employed to remove nonspecific background staining and enhance contrast. After rinsing with distilled water, the sections were stained with Eosin solution (ZSGB-BIO, China) for 3 min, followed by dehydration with graded alcohols and clearing in xylene. Images were captured using a microscope (Leica DMI3000B) and observed at a magnification of ×20.

### Immunohistochemical (IHC) staining

Tissue sections were subjected to antigen retrieval by incubating in Tris-EDTA antigen retrieval buffer (C1038, Solarbio) at 95 ^∘^C for 10 min, followed by endogenous peroxidase blocking at room temperature for 10 min. Subsequently, nonspecific binding was blocked with goat serum at room temperature for 30 min. The sections were then incubated overnight at room temperature with primary antibodies SPINK4, HK2, GLUT1, LDHA, and PKM2 at a 1:1000 dilution. Following primary antibody incubation, the sections were incubated with goat anti-rabbit immunoglobulin G (IgG) secondary antibody at room temperature for 1 h, followed by incubation with HRP-conjugated avidin-biotin complex for 15 min. Immunoreactivity was visualized using the DAB substrate kit (ZSGB, China). Subsequently, the sections were counterstained with 1% hematoxylin. Images were captured using a microscope (Leica DMI3000B), and the staining intensity of positively stained cells was assessed.

### Ethical statement

All animal experiments in this study were conducted in compliance with the “Regulations for the Administration of Affairs Concerning Experimental Animals” (China) and adhered to the ethical guidelines provided in the “Guidelines for the Care and Use of Laboratory Animals” (China). This study was approved by the Animal Welfare and Ethics Group, Department of Laboratory Animal Science, Fudan University (Approval Number: 2023JS MZX-313). Nude mice were purchased from Fudan University, and all experimental procedures complied with the guidelines and standards of the Animal Welfare and Ethics Group of the Department of Laboratory Animal Science, Fudan University. All necessary measures were taken to minimize animal suffering and ensure animal welfare.

### Statistical analysis

The statistical analysis was performed using the R language package (version 4.0.3). Each experiment was repeated three times and the mean ± standard deviation (SD) of the data is presented. One-way analysis of variance (ANOVA) was used, and Tukey’s post hoc test was conducted for multiple comparisons. A *P* value less than 0.05 indicates statistical significance.

## Results

### Analysis of *SPINK4* expression in CRC and its association with patient prognosis

**Figure 1.**
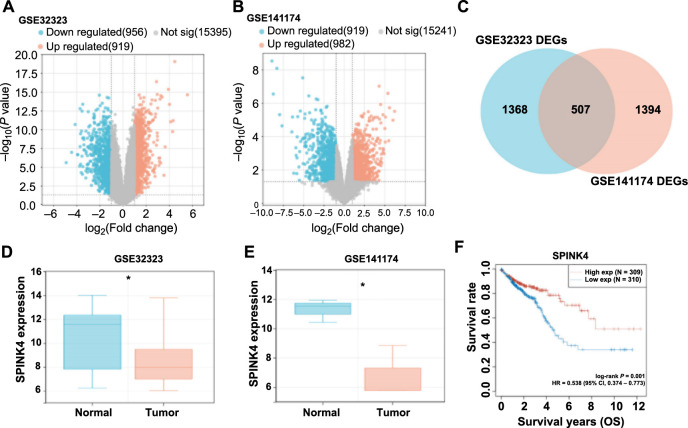
**Analysis of *SPINK4* expression and survival in CRC patients.** (A and B) Volcano plots of filtered DEGs of GSE32323 and GSE141174 datasets. Orange represents upregulated DEGs, blue represents downregulated DEGs, and gray represents insignificant genes; (C) The Venn diagram obtained by intersecting the DEGs of the GSE32323 data set and the GSE141174 data set and the DEGs of the GSE141174 data set; (D and E) Differential expression of *SPINK4* in normal samples and tumor samples of GSE32323 and GSE141174 datasets, respectively. Orange represents tumor samples and blue represents normal samples; (F) The impact of differential expression of *SPINK4* on OS prognosis in CRC patients. Red represents *SPINK4*. *P < 0.05. OS: Overall survival; HR: Hazard ratio; 95% CI: 95% confidence interval of the estimated HR; CRC: Colorectal cancer; DEGs: Differentially expressed genes; SPINK4: Serine peptidase inhibitor, Kazal type 4.

In this study, we employed the R package to screen 919 upregulated DEGs and 956 downregulated DEGs from the GSE32323 dataset (Figure 1A). Additionally, from the GSE141174 dataset, 982 upregulated DEGs and 919 downregulated DEGs were identified (Figure 1B). Through the bioinformatics platform, 507 overlapping genes were identified from the DEGs of both datasets, indicating the existence of common regulatory mechanisms (Figure 1C). Among these overlapping genes, *SPINK4* showed significant downregulation in tumor samples of GSE32323 and GSE141174 datasets (Figure 1D and 1E), and the findings of the survival study demonstrated that increased *SPINK4* expression was linked to good prognosis of CRC patients (Figure 1F), highlighted the possible contribution of *SPINK4* in CRC development.

### Overexpression of *SPINK4* inhibits the proliferation, migration, and invasion of CRC cells

**Figure 2.**
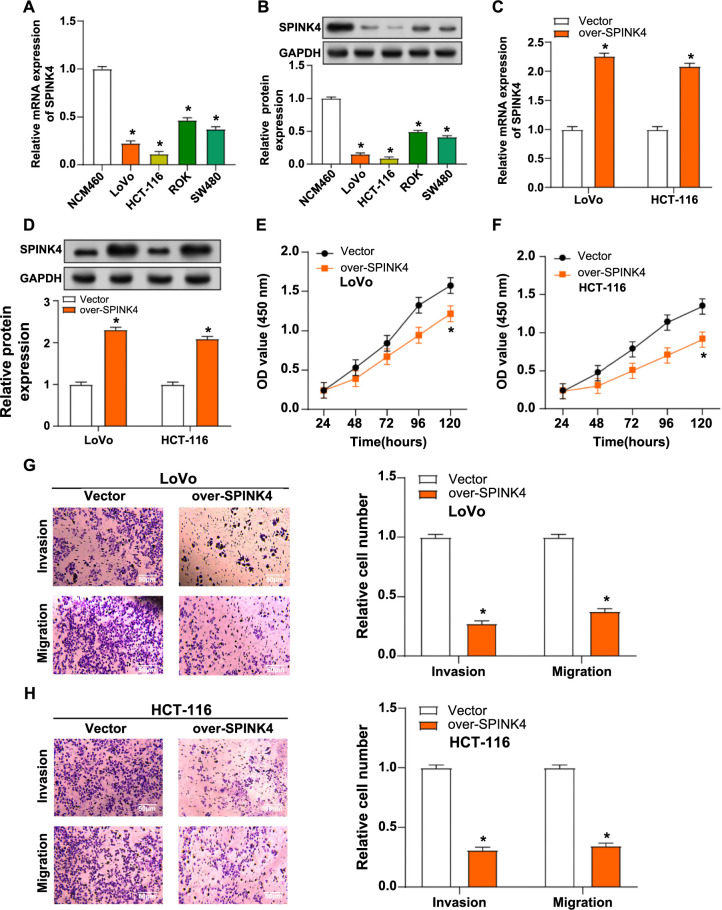
***SPINK4* overexpression inhibits the proliferation, invasion, and migration of CRC cells.** (A and B) qRT-PCR and WB detected the expression of *SPINK4* in normal cell lines (NCM480) and CRC cell lines (HCT116, LoVo, RKO, and SW480); (C and D) qRT-PCR and WB detected the overexpression efficiency of *SPINK4* in LoVo and HCT-116 cells; (E and F) CCK-8 assay detects the proliferation of LoVo and HCT-116 cells after overexpression of *SPINK4*; (G and H) Transwell assay detection of invasion and migration of LoVo and HCT-116 cells after overexpression of *SPINK4*. Orange represents the overexpression of *SPINK4*. *P < 0.05. WB: Western blotting; CRC: Colorectal cancer; CCK-8: Cell counting kit-8; qRT-PCR: Quantitative real-time polymerase chain reaction; SPINK4: Serine peptidase inhibitor, Kazal type 4.

qRT-PCR revealed a significant downregulation of SPINK4 expression in CRC cell lines compared to normal colorectal epithelial cells. This finding was further confirmed by WB analysis, demonstrating a corresponding decrease in protein levels of the gene (Figure 2A and 2B). Notably, among the CRC cell lines, HCT-116 and LoVo exhibited the lowest *SPINK4* expression levels, prompting their selection for subsequent experiments. After identifying low *SPINK4* expression, overexpression experiments were conducted, and overexpression efficiency was assessed (Figure 2C and 2D). CCK-8 assays demonstrated a significant reduction in proliferative activity upon overexpression of *SPINK4* in CRC cells (Figure 2E and 2F). Consistent with the CCK-8 results, Transwell assays revealed a notable decrease in invasion and migration capabilities in CRC cells overexpressing *SPINK4* (Figure 2G and 2H). These findings collectively suggested that the upregulation of *SPINK4* exerts suppressive effects on the proliferation, migration, and invasion abilities of CRC cells.

### Knockdown of *SPINK4* promotes proliferation, invasion, and migration of CRC cells

**Figure 3.**
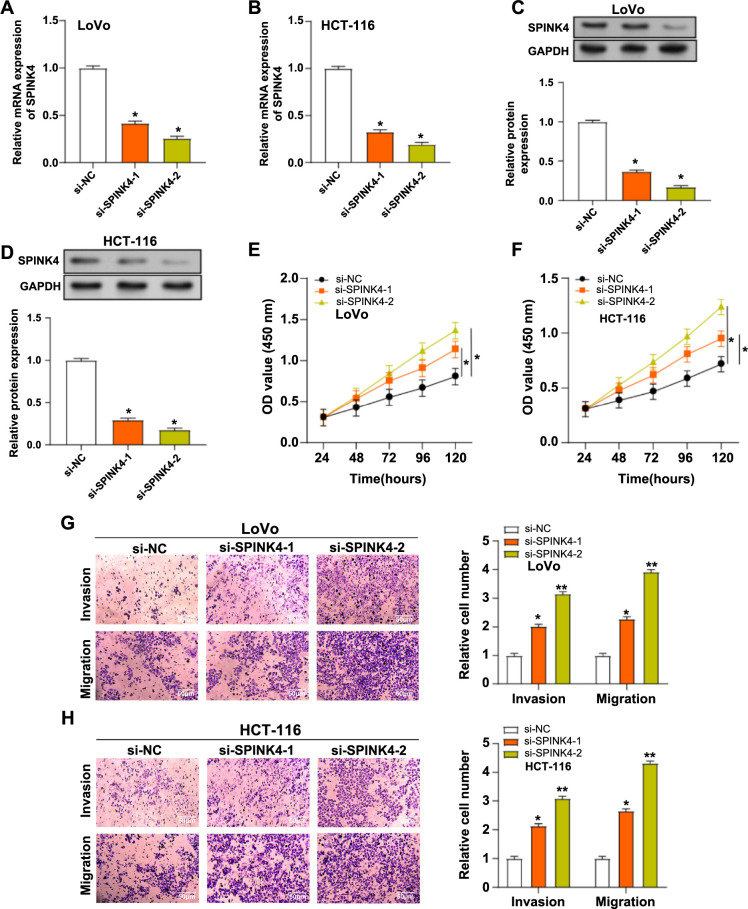
**Knockdown of *SPINK4* promotes the proliferation, invasion, and migration of CRC cells.** (A–D) qRT-PCR and WB detected the knockdown efficiency of *SPINK4* in LoVo and HCT116 cells. Orange represents si-*SPINK4*-1, and green represents si-*SPINK4*-2; (E and F) CCK-8 detects the proliferation of LoVo and HCT-116 cells after knocking down *SPINK4*. Orange represents si-*SPINK4*-1, and green represents si-*SPINK4*-2; (G and H) Transwell detection of invasion and migration of LoVo and HCT-116 cells after knocking down *SPINK4*. Orange represents si-*SPINK4*-1, and green represents si-*SPINK4*-2. *P < 0.05, **P < 0.01. WB: Western blotting; CRC: Colorectal cancer; CCK-8: Cell counting kit-8; qRT-PCR: Quantitative real-time polymerase chain reaction; SPINK4: Serine peptidase inhibitor, Kazal type 4.

qRT-PCR and WB analyses confirmed the effective knockdown of *SPINK4* in CRC cells with both si-*SPINK4*-1 and si-*SPINK4*-2 (Figure 3A–3D). Notably, si-*SPINK4*-2 exhibited superior knockdown efficiency compared to si-*SPINK4*-1. Subsequent functional assays, including CCK-8 and Transwell, showed that knockdown of *SPINK4* with si-*SPINK4*-1 and si-*SPINK4*-2 enhanced the capacity of CRC cells to proliferate, migrate, and invade dramatically (Figure 3E–3H). Interestingly, si-*SPINK4*-2 demonstrated a more pronounced enhancement in cell proliferation, migration, and invasion capacities, prompting its selection for further experimental investigations.

### Knockdown of *SPINK4* induces CRC cell cycle arrest

**Figure 4.**
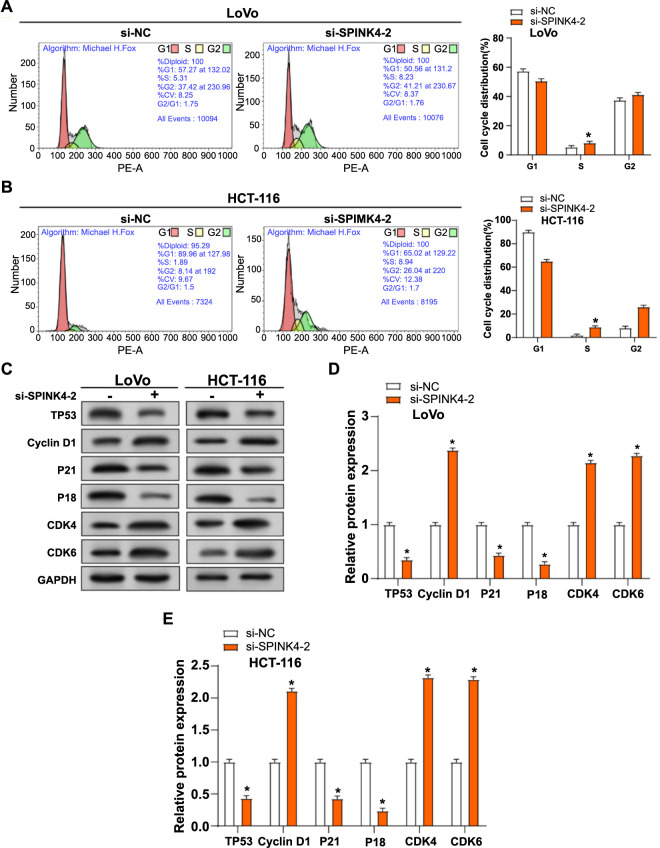
**si-*SPINK4*-2 induces CRC cell cycle arrest.** (A and B) Flow cytometry was used to detect the cycle effects of *SPINK4* knockdown on LoVo and HCT-116 cells. Orange represents the knockdown of *SPINK4*; (C–E) WB detection of the expression of related cycle proteins in LoVo and HCT-116 cells after knocking down *SPINK4*. Related cyclins include TP53, Cyclin D1, p21, p18, CDK4 and CDK6. Orange represents the knockdown of *SPINK4*. *P < 0.05. WB: Western blotting; CRC: Colorectal cancer; SPINK4: Serine peptidase inhibitor, Kazal type 4.

Flow cytometry analysis revealed that si-*SPINK4*-2 induction led to G1 phase cell cycle arrest in CRC cells (Figure 4A and 4B). The alterations in the protein levels of important cell cycle regulators were examined using WB analysis. The findings showed that, following *si-SPINK4-2* induction, CRC cells exhibited a considerable rise in Cyclin D1, CDK4, and CDK6, concomitant with a notable decrease in the protein levels of TP53, P21, and P18 in CRC cells (Figure 4C–4E). These findings suggested that *SPINK4*-induced G1 arrest may be closely related to changes in the degrees of expression of important cell cycle proteins.

### *SPINK4* regulates apoptosis and cell proliferation in CRC cells under glucose-limited conditions

**Figure 5.**
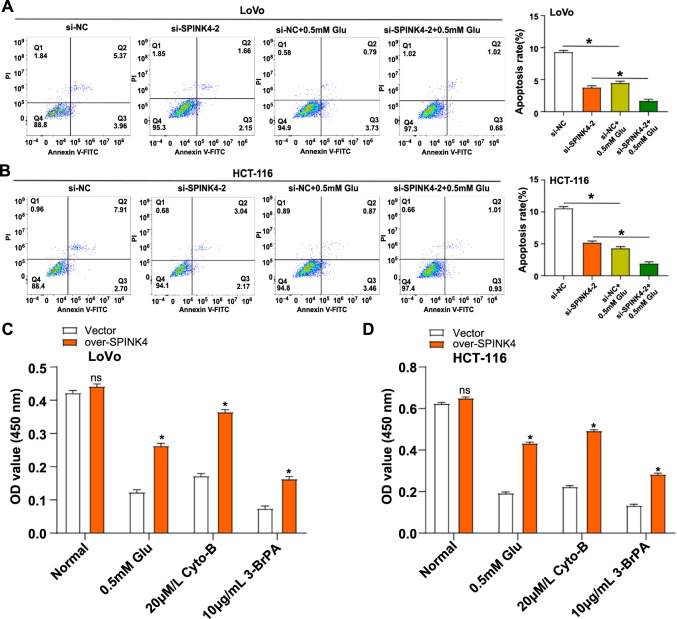
***SPINK4* inhibits aerobic glycolysis in CRC.** (A and B) Flow cytometry was used to detect the apoptosis of LoVo and HCT-116 cells treated with 0.5-mM Glu after knocking down *SPINK4*; (C and D) CCK-8 assay after knocking down *SPINK4*, LoVo, and HCT-116 cells were either cultured under low glucose conditions (0.5-mM glucose), cells were cultured with Cyto-B (20 µM), or cells were treated with 3-BrPA (10 µg/mL) cell viability after 48 h of treatment. ns: Not significant. *P < 0.05. CRC: Colorectal cancer; CCK-8: Cell counting kit-8; SPINK4: Serine peptidase inhibitor, Kazal type 4.

Analysis using flow cytometry revealed that following si-*SPINK4*-2 induction, the apoptotic rate of CRC cells was lowered. Furthermore, under low glucose conditions (0.5 mM Glu), the apoptosis rate of CRC cells was reduced. Interestingly, the combination of si-SPINK4-2 induction and 0.5 mM Glu resulted in a significant further reduction in the apoptotic rate of CRC cells (Figure 5A and 5B). To examine the impact of glucose consumption on cell survival, SPINK4-overexpressing CRC cells were treated with low glucose (0.5 mM Glu), a glucose transport inhibitor (Cyto-B, 20 µM/L), and a hexokinase-2 inhibitor (3-BrPA, 10 µg/mL) for 48 h. The CCK-8 test results showed a notable increase in cell proliferation activity compared to the control group after 48 h of treatment (Figure 5C and 5D). These findings suggest that *SPINK4* regulates CRC cell survival, potentially through its control of apoptotic mechanisms, in a glucose-dependent manner.

### *SPINK4* exerts anti-CRC effects by inhibiting glycolysis

**Figure 6.**
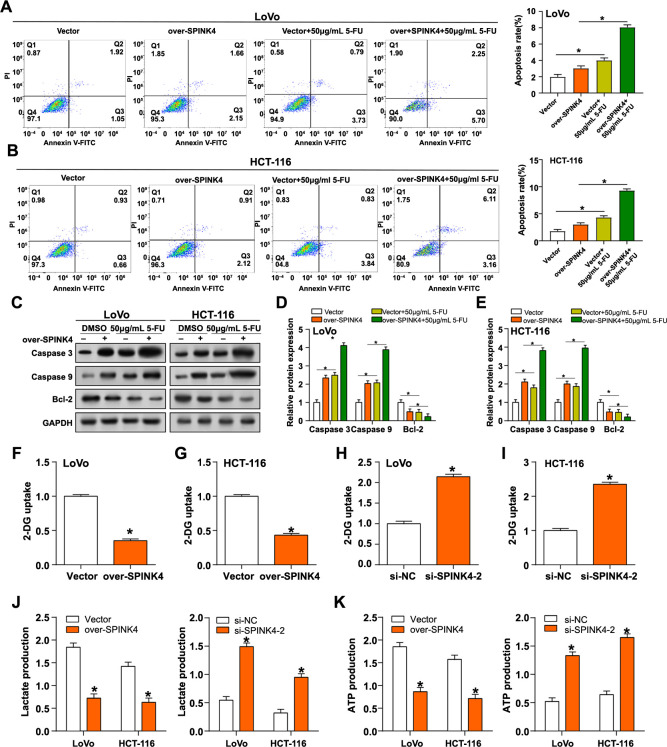
***SPINK4* exerts anti-CRC effects by inhibiting glycolysis.** (A and B) Flow cytometry detects the apoptosis of LoVo and HCT-116 cells with or without (50 µg/mL) 5-FU treatment after overexpressing *SPINK4*; (C–E) WB detected the expression of related apoptotic proteins in LoVo and HCT-116 cells with or without (50 µg/mL) 5-FU treatment after overexpressing *SPINK4*. Related apoptotic proteins include Caspase 3, Caspase 9, and Bcl-2; (F–I) Flow cytometry detects the uptake of 2-DG in LoVo and HCT-116 cells after overexpression or knockdown of *SPINK4*; (J) The lactate detection kit detects lactate release from LoVo and HCT-116 cells after overexpression or knockdown of *SPINK4*; (K) ATP detection kit detects ATP production in LoVo and HCT-116 cells after overexpression or knockdown of *SPINK4*. *P < 0.05. WB: Western blotting; CRC: Colorectal cancer; ATP: Adenosine triphosphate; SPINK4: Serine peptidase inhibitor, Kazal type 4; 2-DG: 2-Deoxy-D-glucose.

5-FU is an anticancer medication that is frequently used to treat different types of cancer. It plays a critical role in cancer treatment, which aims to stop the growth of cancer cells, by compromising DNA integrity, influencing gene expression, and regulating apoptosis. Apoptosis rates of LoVo and HCT-116 cell lines were determined using flow cytometry analysis under different treatment conditions. In LoVo cells, treatment with either overexpression of *SPINK4* or 5-FU (50 µg/mL) alone increased apoptosis compared to vector control. However, apoptosis was significantly increased when both were combined (Figure 6A). In addition, HCT-116 cells showed a similar trend (Figure 6B). Further, WB experiments detected the expression levels of key apoptotic and anti-apoptotic proteins, including Bcl-2, caspase-3, and caspase-9 (Figure 6C–6E). *SPINK4* overexpression results in the upregulation of cleaved (active) forms of caspase-3 and caspase-9 in the presence or absence of 5-FU, indicating activation of the apoptotic pathway. In contrast, *SPINK4* overexpression in conjunction with 5-FU therapy resulted in a downregulation of the levels of the anti-apoptotic protein Bcl-2. In HCT-116 and LoVo cells, overexpression of *SPINK4* significantly reduced uptake of 2-DG, indicating altered glucose metabolism (Figure 6F and 6G). Conversely, overexpression of *SPINK4* led to increased uptake of 2-DG (Figure 6H and 6I). Furthermore, overexpression of *SPINK4* resulted in increased lactate production and ATP levels in both cell lines, indicating a shift toward aerobic glycolysis. Knockdown of *SPINK4* led to the opposite trend, further confirming the effect of this gene on metabolism (Figure 6J and 6K).

### *SPINK4* regulates glycolysis in CRC by inhibiting the β-catenin pathway

**Figure 7.**
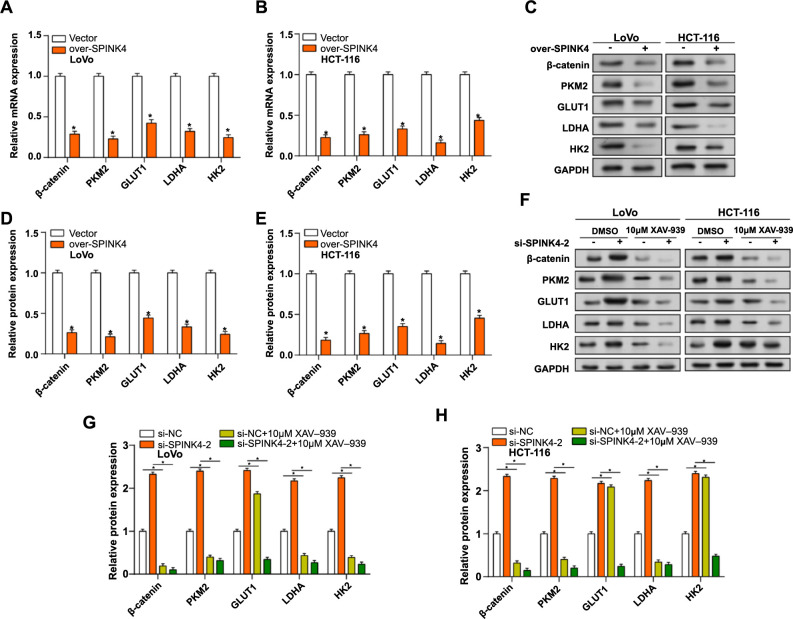
**SPINK4 inhibits glycolysis by affecting β-catenin.** (A–E) qRT-PCR and WB detected the expression of glycolysis-related proteins after overexpression of SPINK4. Glycolysis-related proteins include β-catenin, PKM2, GLUT1, LDHA and HK2; (F–H) WB detection of glycolysis-related protein expression in LoVo and HCT-116 cells with or without β-catenin inhibitor XAV-939 (10 µM, 72 h) after knocking down *SPINK4*. *P < 0.05. WB: Western blotting; qRT-PCR: Quantitative real-time polymerase chain reaction; SPINK4: Serine peptidase inhibitor, Kazal type 4.

The overexpression of *SPINK4* dramatically reduced the expression of glycolytic-related proteins (β-catenin, PKM2, GLUT1, LDHA, and HK2) in CRC cells, as shown by qRT-PCR and WB studies (Figure 7A–7E). Subsequently, the expression of glycolytic-related proteins in CRC cells was evaluated by WB analysis after *SPINK4* knockdown, either with or without the β-catenin inhibitor (XAV-939, 10 µM, 48 h). The findings indicated that induction of si-SPINK4-2 significantly increased the levels of glycolytic-related proteins. However, the addition of 10-µM XAV-939 reversed this elevation, causing a notable reduction in the amounts of glycolytic-related proteins (Figure 7F–7H). These results emphasize the regulatory function of *SPINK4* in modulating the expression of key glycolytic proteins in CRC cells.

### *SPINK4* overexpression inhibits subcutaneous xenograft tumor growth

**Figure 8.**
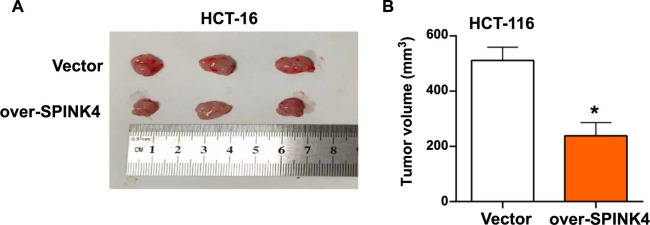
***SPINK4* overexpression inhibits subcutaneous xenograft tumor growth.** (A) Image of subcutaneous xenograft tumor growth. Including the control group and the overexpression *SPINK4* group; (B) Tumor volume after transplantation. Orange represents the overexpression of *SPINK4*. *P < 0.05. SPINK4: Serine peptidase inhibitor, Kazal type 4.

Tumor specimens collected from mice showed that tumor cells overexpressing *SPINK4* were significantly smaller in size compared with vehicle controls (Figure 8A). Quantitative analysis of tumor volume further confirmed this observation, with tumors overexpressing *SPINK4* significantly reduced in size compared with vehicle controls (Figure 8B). These results indicated that *SPINK4* overexpression has an effective inhibitory effect on tumor growth after xenotransplantation.

### SPINK4 enhances glycolytic-related protein expression in CRC

**Figure 9.**
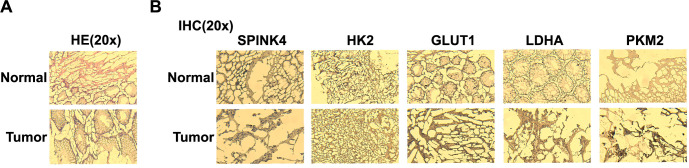
**Increased expression of glycolysis-related proteins in CRC tissues.** (A) Histopathological features of CRC observed through hematoxylin and eosin staining. Representative image showing CRC tissue characteristics. Magnification: ×20; (B) IHC staining of SPINK4, HK2, GLUT1, LDHA, and PKM2 in CRC tissues and normal tissues. Magnification: ×20. SPINK4: Serine peptidase inhibitor, Kazal type 4; HE: Hematoxylin and Eosin; CRC: Colorectal cancer; IHC: Immunohistochemistry.

Histopathological features corresponding to CRC were observed through HE staining (Figure 9A). Compared to normal tissue, tumor samples exhibited disrupted tissue architecture and heterogeneous staining patterns, indicating tumorigenic alterations. Subsequent IHC staining of CRC tissues and normal tissues revealed increased expression of SPINK4, HK2, GLUT1, LDHA, and PKM2 compared to normal tissues (Figure 9B). This further supported the notion that SPINK4 may co-regulate CRC tissues along with glycolysis.

## Discussion

The importance of biomarkers in CRC treatment is crucial. By identifying specific molecular signatures of cancer, biomarkers enable more precise and effective treatment strategies [21]. For example, *NEO1* expression levels decline as CRC advances, and low-expression levels are linked to a poor prognosis, indicating the possibility of *NEO1* as a prognostic marker [22]. Furthermore, functional studies further highlighted the significance of *NEO1* overexpression in therapeutic intervention by demonstrating that it suppressed the proliferation, migration, and invasion of CRC cells. Other studies have also shown that *CREPT* is overexpressed in CRC, contributes to tumor progression and cell proliferation, is positively correlated with the efficacy of 5-FU treatment, and can serve as a valuable prognostic biomarker for CRC patients [23]. Current diagnostic modalities, targeted gene therapies, and five-year survival rates from CRC have been key aspects of research. This discussion highlights the search for more effective biomarkers for the diagnosis, treatment, and prognosis of CRC. The exploration of key biomarkers not only improves diagnostic accuracy but also holds the promise of improving treatment strategies and predicting patient outcomes. In this study, based on our bioinformatics analysis, we found that *SPINK4* showed downregulation in CRC and was linked to a favorable prognosis for CRC patients. This observation provides valuable molecular insights into dysregulated processes associated with CRC and highlights the need for further studies to elucidate the functional implications of *SPINK4* alterations and their potential as a diagnostic or prognostic indicator for CRC.

The importance of *SPINK4* in previous studies on cancer also lies in its involvement in tumor proliferation, migration, and invasion [24]. This multifaceted role emphasizes the key impact of *SPINK4* on the invasiveness of cancer cells, further emphasizing its role in oncology research. Supported by the research results of Li et al. [25], the increased expression of *SPINK4* in CRC is related to HCT-116 and DXH-1 cell invasion, migration, proliferation, and cell cycle progression. ELF-1, binding to the *SPINK4* promoter, influenced its expression, and elevated ELF-1 levels were associated with poorer prognosis in colon cancer. Suppression of *SPINK4* counteracted ELF-1 overexpression effects, suggesting a therapeutic avenue targeting ELF-1/*SPINK4* expression for colon cancer treatment. Correspondingly, Hu et al. [26] demonstrated that *SPINK4* downregulation in CRC tissues was linked to enhanced proliferation and metastasis, while *SPINK4* overexpression promoted CRC cell proliferation, metastasis, and tumor growth, primarily localized in the nucleoplasm and nucleus. Moreover, *SPINK4* inhibited ferroptosis in CRC cells, emphasizing its multifaceted role in CRC pathogenesis by influencing cell proliferation, metastasis, and ferroptosis. Our in vitro studies revealed that downregulation of *SPINK4* stimulates these activities and causes cell cycle stasis in CRC cells during the G1 phase, whereas overexpression of *SPINK4* blocks the proliferation, invasion, and migration of CRC cells. Together, these results highlight the potential of *SPINK4* as a therapeutic target for CRC treatment approaches and imply that it plays a dual function in CRC.

Glucose, cytochalasin B (Cyto-B), and 3-bromopyruvate (3-BrPA) are related to each other through their effects on cellular processes [27, 28]. Glucose, a fundamental carbohydrate and primary energy source, is central to cellular metabolism. Cyto-B disrupts actin polymerization, influencing cellular structure, while 3-BrPA inhibits glycolysis, a key metabolic pathway involving glucose breakdown. Their applications in biological studies and therapeutic interventions stem from their effects on cellular functions and metabolism. Subsequently, exemplifying the role of glucose in CRC, the research of Zhu et al. [29] demonstrates that ketone bodies in CRC cells modulate MondoA phosphorylation, affecting glucose uptake, apoptosis, and proliferation, with MEK1 identified as a novel influencer. Zhu et al. [30]explores a novel approach, combining glucose oxidase and gold nanorods in a nanoreactor for colon cancer treatment. Meanwhile, the study of Zhou et al. [31] highlights Glucose-regulated protein 78 (GRP78) overexpression in CRC, impacting cancer cell proliferation, tumorigenesis, and resistance to 5-FU through AKT and ERK pathways, establishing a significant association between CRC progression and glucose regulation. In conclusion, our research findings indicate that *SPINK4* inhibits aerobic glycolysis in CRC, providing valuable insights into potential therapeutic strategies for CRC treatment.

5-FU is a chemotherapeutic agent used in cancer treatment, inhibiting thymidylate synthase and disrupting DNA synthesis. A glucose analog, 2-DG, interferes with glycolysis, impacting cellular energy metabolism. Lactic acid (lactate) is a byproduct of anaerobic glycolysis, produced when cells generate energy without oxygen. ATP is the cell’s primary energy currency, generated through various cellular processes, including glycolysis and oxidative phosphorylation. The relationship between these compounds lies in their involvement in cellular metabolism and energy production. 5-FU disrupts DNA synthesis, affecting cell division [32]. 2-DG interferes with glycolysis, reducing glucose utilization and ATP production [33]. Lactic acid is produced as a result of anaerobic glycolysis, and ATP is the end product of cellular energy metabolism, essential for various cellular functions [34]. These compounds are interconnected through their roles in cellular metabolism and energy production, impacting CRC progression. Zykova et al. [35] demonstrated that elevated expressions of phosphorylated p53-related protein kinase (p-PRPK) in colon adenocarcinomas correlate with advanced metastatic stages. PRPK, a known phosphorylator of survivin, promotes colon cancer metastasis, suggesting its potential as a prognostic marker. Fusidic acid, an FDA-approved antibiotic, when combined with 5-FU, inhibits PRPK activity, providing an alternative therapeutic strategy for colon cancer patients. Additionally, Zupi et al. [36] found that the combination of N-methylformamide (NMF) with 5-FU significantly enhances cytotoxicity in human colon cancer cells (HT29), with the 5-FU→NMF sequence demonstrating a potent decrease in cell survival. Furthermore, the study of Zou ZW et al. revealed that Connexin43 (Cx43) gap junctions play a role in modulating CRC cells’ resistance to 5-FU, oxaliplatin, and irinotecan provide a potential basis for therapeutic strategies to overcome drug resistance in colon cancer [37]. Meanwhile, Zou et al. [38] reported that insulin pretreatment significantly augments the anticancer functions of 5-FU in colonic (Ls-174-t) and human esophageal (Eca 109) cancer cells, leading to increased apoptosis, altered cell cycle distribution, and enhanced inhibition of cell proliferation. At the same time, our study found that *SPINK4* exerts anti-CRC effects by inhibiting glycolysis. In conclusion, the diverse roles of 5-FU, 2-DG, lactate, and ATP in CRC metabolism and treatment strategies underscore their potential impact on cellular processes and cancer progression.

Glycolysis-related proteins play crucial roles in cellular energy metabolism. The multifunctional protein β-catenin can affect the expression of glycolytic genes and is implicated in Wnt signaling [39]. Pyruvate kinase M2, or PKM2, controls the last stage of glycolysis and encourages aerobic glycolysis in cancer cells [40]. Cells are aided in absorbing glucose by the GLUT1. LDHA converts pyruvate to lactate to aid in anaerobic glycolysis [41]. Glycolysis is started by phosphorylating glucose by HK2. It is possible to gain a better understanding of the complex regulation of glycolysis and its potential therapeutic uses in many diseases, including cancer, by understanding the functions and interactions of these proteins. Research on *CD36* and *NDRG2* provides crucial information on glycolysis-related proteins in CRC. Found by Xu et al. [42], *NDRG2* suppresses glycolysis and glutaminolysis in CRC cells by blocking c-Myc via β-catenin mediation. In addition, CD36, as described by Fang et al. [43], inhibits GPC4 ubiquitination and β-catenin/c-myc signaling to reduce glycolysis as a tumor suppressor in CRC. Both results illustrate the intricate regulatory networks that include *CD36* and *NDRG2*, as well as their roles in regulating metabolic pathways and safeguarding against cancer. Due to our research, β-catenin is regulated by *SPINK4* and inhibits glycolysis. Notably, xenograft research demonstrates that *SPINK4* can prevent CRC cell growth in vivo. After HE staining, the histopathological features of CRC tissues were revealed, while IHC staining exhibited elevated expression levels of SPINK4, HK2, GLUT1, LDHA, and PKM2 within CRC tissues. All of these findings contributed to our growing knowledge of the complex dynamics of glycolytic regulation and emphasized the potential use of these pathways in the treatment of CRC.

Despite the valuable insights gained from our study, it is important to acknowledge that there are several limitations that may affect the interpretation and generalization of our findings. Our study was constrained by limited resources, including funding and specialized equipment. This limitation hindered our ability to perform exhaustive experiments and additional analyses such as the disclosure of whole xenograft samples and histological examinations such as HE staining and immunohistochemical (IHC) of representative proteins. The time-limited nature of our study limits the depth to which we can investigate complex mechanisms. As a result, certain aspects may have been overlooked, thus limiting our full understanding of the regulatory role of SPINK4. Although our study provides compelling evidence that SPINK4 regulation is involved in CRC progression, it remains a preliminary exploration. The intricacies of the SPINK4-mediated signaling pathways need to be further elucidated through advanced experimental techniques and interdisciplinary collaborations. In conclusion, despite the substantial insights gained from our study, its limitations must be acknowledged. Addressing these limitations in future research endeavors will pave the way for a deeper understanding of the regulatory mechanisms of SPINK4 in CRC.

## Conclusion

In conclusion, a detailed analysis of *SPINK4* in CRC showed that it is markedly downregulated in tumor tissues and is associated with a poor prognosis. Functional analysis demonstrates that SPINK4 acts as a tumor suppressor by inhibiting the growth, invasion, and migration of CRC cells and inducing cell cycle arrest in the G1 phase. Notably, it was shown that glucose availability influenced how it affected cell proliferation and that *SPINK4* decreased apoptosis in CRC cells. It is significant to note that *SPINK4* inhibited glycolysis to produce ATP, change lactate release, and alter 2-DG absorption, all of which were indicators of its anti-CRC activities. Mechanistically, SPINK4 modulated the level of glycolytic-related proteins, including β-catenin, PKM2, LDHA, GLUT1, and HK2. Additionally, *SPINK4* overexpression suppressed subcutaneous xenograft tumor growth in vivo. These findings underscored the intricate regulatory role of *SPINK4* in CRC, shedding light on its potential as a therapeutic target for inhibiting tumorigenesis and modulating glycolytic pathways in CRC patients.

## Data Availability

The datasets used and/or analyzed during the current study are available from the corresponding author on reasonable request.

## References

[1] Testa U, Pelosi E, Castelli G (2018). Colorectal cancer: genetic abnormalities, tumor progression, tumor heterogeneity, clonal evolution and tumor-initiating cells. Med Sci.

[2] Wang H, Tian T, Zhang J (2021). Tumor-associated macrophages (TAMs) in colorectal cancer (CRC): from mechanism to therapy and prognosis. Int J Mol Sci.

[3] Murphy N, Moreno V, Hughes DJ, Vodicka L, Vodicka P, Aglago EK (2019). Lifestyle and dietary environmental factors in colorectal cancer susceptibility. Mol Aspects Med.

[4] van der Sloot KW, Amini M, Peters V, Dijkstra G, Alizadeh BZ (2017). Inflammatory bowel diseases: review of known environmental protective and risk factors involved. Inflam Bowel Dis.

[5] Sawicki T, Ruszkowska M, Danielewicz A, Niedźwiedzka E, Arłukowicz T, Przybyłowicz KE (2021). A review of colorectal cancer in terms of epidemiology, risk factors, development, symptoms and diagnosis. Cancers.

[6] Hossain MS, Karuniawati H, Jairoun AA, Urbi Z, Ooi DJ, John A (2022). Colorectal cancer: a review of carcinogenesis, global epidemiology, current challenges, risk factors, preventive and treatment strategies. Cancers.

[7] Li J, Ma X, Chakravarti D, Shalapour S, DePinho RA (2021). Genetic and biological hallmarks of colorectal cancer. Genes Develop.

[8] Ponomaryova AA, Rykova EY, Solovyova AI, Tarasova AS, Kostromitsky DN, Dobrodeev AY (2023). Genomic and transcriptomic research in the discovery and application of colorectal cancer circulating markers. Int J Mol Sci.

[9] Pellino G, Gallo G, Pallante P, Capasso R, De Stefano A, Maretto I (2018). Noninvasive biomarkers of colorectal cancer: role in diagnosis and personalised treatment perspectives. Gastroenterol Res Pract.

[10] Kalyanaraman B (2017). Teaching the basics of cancer metabolism: developing antitumor strategies by exploiting the differences between normal and cancer cell metabolism. Redox Biol.

[11] Chandel NS (2021). Glycolysis. Cold Spring Harb Perspect Biol.

[12] Dorlon J. https://scholar.google.com/scholar?hl=zh-CN&as_sdt=0%2C5&q=Dorlon+J.+Metabolisms.+Microreviews+in+Cell+and+Molecular+Biology.+2021%3B8%282%29.&btnG=.

[13] Zuo S, Wu L, Wang Y, Yuan X (2020). Long non-coding RNA MEG3 activated by vitamin D suppresses glycolysis in colorectal cancer via promoting c-Myc degradation. Front Oncol.

[14] Zhu Y, Shi T, Lu X, Xu Z, Qu J, Zhang Z (2021). Fungal-induced glycolysis in macrophages promotes colon cancer by enhancing innate lymphoid cell secretion of IL-22. EMBO J.

[15] Zhu W, Zhou B-L, Rong L-J, Ye L, Xu H-J, Zhou Y (2020). Roles of PTBP1 in alternative splicing, glycolysis, and oncogensis. J Zhejiang Univ Sci B.

[16] Kumaresan V, Harikrishnan R, Arockiaraj J (2015). A potential Kazal-type serine protease inhibitor involves in kinetics of protease inhibition and bacteriostatic activity. Fish Shellfish Immunol.

[17] Chen T-J, Tian Y-F, Chou C-L, Chan T-C, He H-L, Li W-S (2021). High SPINK4 expression predicts poor outcomes among rectal cancer patients receiving CCRT. Current Oncol.

[18] Xie M, Li K, Li J, Lu D, Hu B (2019). Association and diagnostic value of serum SPINK4 in colorectal cancer. PeerJ.

[19] Wang X, Yu Q, Ghareeb WM, Zhang Y, Lu X, Huang Y (2019). Downregulated SPINK4 is associated with poor survival in colorectal cancer. BMC cancer.

[20] Buijs JT, van Beijnum R, Anijs RJ, Laghmani EH, Sensuk L, Minderhoud C (2023). The association of tumor-expressed REG4, SPINK4 and alpha-1 antitrypsin with cancer-associated thrombosis in colorectal cancer. J Thromb Thrombol.

[21] Koncina E, Haan S, Rauh S, Letellier E (2020). Prognostic and predictive molecular biomarkers for colorectal cancer: updates and challenges. Cancers.

[22] Zhang M, Zhou Z, Pan X-K, Zhou Y-J, Li H-O, Qiu P-S (2020). Identification of NEO1 as a prognostic biomarker and its effects on the progression of colorectal cancer. Cancer Cell Int.

[23] Kuang Y-S, Wang Y, Ding L-D, Yang L, Wang Y, Liu S-H (2018). Overexpression of CREPT confers colorectal cancer sensitivity to fluorouracil. World J Gastroenterol.

[24] Liao C, Wang Q, An J, Zhang M, Chen J, Li X (2022). Spinks in tumors: potential therapeutic targets. Front Oncol.

[25] Li T, Jia Z, Liu J, Xu X, Wang H, Li D (2023). Transcription activation of SPINK4 by ELF-1 augments progression of colon cancer by regulating biological behaviors. Tissue Cell.

[26] Hu B-L, Yin Y-X, Li K-Z, Li S-Q, Li Z (2023). SPINK4 promotes colorectal cancer cell proliferation and inhibits ferroptosis. BMC Gastroenterol.

[27] McDaniel M, King S, Anderson S, Fink J, Lacy P (1974). Effect of cytochalasin B on hexose transport and glucose metabolism in pancreatic islets. Diabetologia.

[28] Sun X, Sun G, Huang Y, Hao Y, Tang X, Zhang N (2020). 3-Bromopyruvate regulates the status of glycolysis and BCNU sensitivity in human hepatocellular carcinoma cells. Biochem Pharmacol.

[29] Zhu Y, Xu N, Wu S, Luan Y, Ke H, Wu L (2023). MEK1-dependent MondoA phosphorylation regulates glucose uptake in response to ketone bodies in colorectal cancer cells. Cancer Sci.

[30] Zhu H, Li Y, Ming Z, Liu W (2021). Glucose oxidase-mediated tumor starvation therapy combined with photothermal therapy for colon cancer. Biomater Sci.

[31] Zhou X, Xing X, Zhang S, Liu L, Wang C, Li L (2016). Glucose-regulated protein 78 contributes to the proliferation and tumorigenesis of human colorectal carcinoma via AKT and ERK pathways. Oncol Rep.

[32] Hu J, Li A, Guo Y, Ma T, Feng S (2023). The relationship between tumor metabolism and 5-fluorouracil resistance. Biochem Pharmacol.

[33] Pajak B, Siwiak E, Sołtyka M, Priebe A, Zieliński R, Fokt I (2019). 2-Deoxy-d-glucose and its analogs: from diagnostic to therapeutic agents. Int J Mol Sci.

[34] Melkonian EA, Schury MP (2019). Biochemistry, anaerobic glycolysis [Internet]. Treasure Island (FL): StatPearls Publ;.

[35] Zykova T, Zhu F, Wang L, Li H, Lim DY, Yao K (2018). Targeting PRPK function blocks colon cancer metastasis. Mol Cancer Ther.

[36] Zupi G, Marangolo M, Arancia G, Greco C, Laudonio N, Iosi F (1988). Modulation of the cytotoxic effect of 5-fluorouracil by N-methylformamide on a human colon carcinoma cell line. Cancer Res [Internet].

[37] Zou ZW, Chen HJ, Yu JL, Huang ZH, Fang S, Lin XH (2016). Gap junction composed of connexin43 modulates 5-fluorouracil, oxaliplatin and irinotecan resistance on colorectal cancers. Mol Med Rep.

[38] Zou K, JU Jh, Xie H (2007). Pretreatment with insulin enhances anticancer functions of 5-fluorou-racil in human esophageal and colonic cancer cells. Acta Pharmacologica Sinica.

[39] Zuo Q, He J, Zhang S, Wang H, Jin G, Jin H (2021). PPARγ Coactivator-1α suppresses metastasis of hepatocellular carcinoma by inhibiting Warburg effect by PPARγ–Dependent WNT/β-Catenin/Pyruvate Dehydrogenase Kinase Isozyme 1 Axis. Hepatology.

[40] Zahra K, Dey T, Ashish, Mishra SP, Pandey U (2020). Pyruvate kinase M2 and cancer: the role of PKM2 in promoting tumorigenesis. Front Oncol.

[41] Berg K. http://urn.nb.no/URN:NBN:no--97158.

[42] Xu X, Li J, Sun X, Guo Y, Chu D, Wei L (2015). Tumor suppressor NDRG2 inhibits glycolysis and glutaminolysis in colorectal cancer cells by repressing c-Myc expression. Oncotarget.

[43] Fang Y, Shen Z-Y, Zhan Y-Z, Feng X-C, Chen K-L, Li Y-S (2019). CD36 inhibits β-catenin/c-myc-mediated glycolysis through ubiquitination of GPC4 to repress colorectal tumorigenesis. Nat Commun.

